# Body mass index mediates the association between serum fibroblast growth factor-19 and diabetes

**DOI:** 10.1007/s40200-025-01850-y

**Published:** 2026-01-08

**Authors:** Lin Liu, Shuyun Liu, Juanying Zhen, Lijie Ren, Guoru Zhao, Jianguo Liang, Aimin Xu, Chao Li, Jun Wu, Bernard MY Cheung

**Affiliations:** 1 Department of Medicine, School of Clinical Medicine, The University of Hong Kong, Hong Kong, China; 2 Department of Neurology, Peking University Shenzhen Hospital, Shenzhen, China; 3 Department of Neurology, Shenzhen Longhua District Central Hospital, Shenzhen, China; 4 Department of Neurology, The First Affiliated Hospital of Shenzhen University, Shenzhen Second People’s Hospital, Shenzhen, China; 5 CAS Key Laboratory of Human-Machine Intelligence-Synergy Systems, Research Center for Neural Engineering, Shenzhen Institute of Advanced Technology, Chinese Academy of Sciences, Shenzhen, China; 6 Precision Health Research Center Company Limited, Hong Kong, SAR China; 7 State Key Laboratory of Pharmaceutical Biotechnology, The University of Hong Kong, Pokfulam, Hong Kong, China; 8 Institute of Cardiovascular Science and Medicine, The University of Hong Kong, Pokfulam, Hong Kong, China

**Keywords:** Fibroblast growth factor, Diabetes, Body mass index, Mediation

## Abstract

**Background:**

This study aimed to investigate the relationship between serum fibroblast growth factor-19 (FGF19) levels and diabetes, as well as the potential mediating role of body mass index (BMI).

**Methods:**

Data from 1,018 participants with valid serum FGF19 measurements from the Shenzhen–Hong Kong United Network on Cardiovascular Disease study were analyzed. Logistic regression models were used to estimate odds ratios (ORs) and 95% confidence intervals (CIs) for diabetes risk. Mediation analysis was conducted to assess the extent to which BMI mediated the association between serum FGF19 levels and diabetes.

**Results:**

The mean age of participants was 45.1 years, with 77 individuals (7.6%) diagnosed with diabetes. Natural logarithm (ln)-transformed FGF19 levels were inversely associated with BMI [age- and sex-adjusted β (95% CI): -0.30 (-0.58, -0.03)]. Ln-transformed FGF19 levels were also negatively associated with diabetes after adjusting for covariates, including age, sex, smoking status, alcohol consumption, blood pressure, BMI, waist circumference, and lipid profile [OR (95% CI): 0.66 (0.47–0.95)]. No significant interactions were observed between ln-transformed FGF19 levels and age, sex, obesity, or abdominal obesity in relation to diabetes. Mediation analysis revealed that BMI reduction accounted for 10.1% of the association between ln-transformed FGF19 levels and diabetes.

**Conclusion:**

Serum FGF19 levels are inversely associated with diabetes risk in the Chinese population, with BMI serving as a partial mediator of this relationship.

**Supplementary Information:**

The online version contains supplementary material available at 10.1007/s40200-025-01850-y.

## Introduction

Diabetes is a significant global public health concern, with a prevalence of 8.5% among adults over 18 years in 2019 and projected to increase to 700 million by 2045 [1]. This complex disease has a multifactorial etiology and is associated with an elevated risk of cardiovascular disease and imposes a considerable financial burden on healthcare systems, particularly in low- and middle-income countries [2].

Fibroblast growth factor 19 (FGF19) is a hormone produced primarily in the intestine, in response to bile acids, and plays a vital role in lipid and carbohydrate metabolism [3]. In patients with both obesity and type 2 diabetes, these two common metabolic disorders share some pathophysiology, which was associated with decreased fasting plasma levels of FGF19 [4]. FGF19 analogs show potential for antidiabetic effects and weight loss, with proven efficacy in mice [5, 6]. However, a clinical trial showed it had only a modest effect on reducing body weight, and it failed to reduce hyperglycemia [7]. To clarify the overlapping association between diabetes, body mass index (BMI), and serum FGF19, this study aimed to investigate the relationship between serum FGF19 and diabetes in the Chinese population and estimate the potential mediating effect of BMI.

## Method

### Study population

This study utilized data from the Shenzhen–Hong Kong United Network on Cardiovascular Disease (SHUN-CVD) and details of the study can be found elsewhere [8]. We enrolled 1018 participants with valid data on serum FGF19 levels and diabetes, with the flow chart shown in Figure S1. The study was reviewed and approved by the institutional review board of Peking University Shenzhen Hospital and the University of Hong Kong. All enrolled participants have provided written informed consent.

### Measurement and definition

Structured questionnaires were used to collect demographic data, medical history, medication use, alcohol consumption, and smoking status. Trained examiners conducted physical examinations, including systolic blood pressure (BP), diastolic BP, body weight and height, and waist circumference (WC). Blood samples were taken after a minimum 8-hour fasting period for fasting blood glucose (FBG), hemoglobin A1c (HbA1c), lipid profile, and high sensitivity C-Reactive Protein (hs-CRP). The level of FGF19 was measured using ELISA kits supplied by ImmunoDiagnostics Limited (https://www.immunodiagnostics.com.hk/product-page/human-fgf-19-elisa-kit). The lowest level of FGF-19 that can be measured by this assay is 31.2 pg/mL. The antibodies used in this assay are specific to human FGF-19 and do not cross-react with human Adiponectin, FGF-21, Fatty Acid Binding Protein 4 (FABP4), Lipocalin-2 (LCN2), Retinol Binding Protein 4 (RBP4) and Plasminogen Activator Inhibitor-1 (PAI-1). Intra-assay and inter-assay coefficients of variation are < 4.5%, and < 5.6%, respectively. Current smoking was defined as the daily smoking of any tobacco product, and participants were considered drinkers if they consumed any type of alcoholic beverage at least once a week. BMI (kg/m^2^) was calculated as weight (in kilograms) divided by height (in meters squared). BMI from 24 to 28 kg/m^2^ was defined as overweight and BMI ≥ 28 kg/m^2^ was defined as obesity according to the criteria recommended for Chinese adults. Central obesity was defined as a WC ≥ 90 cm in males or a WC ≥ 85 cm in females. Hypertension was defined as systolic BP ≥ 140 mm Hg or diastolic BP ≥ 90 mm Hg, or the use of anti-hypertensive medication. Diabetes was defined as fasting blood glucose ≥ 126 mg/dL or HbA1c ≥ 6.5%, or the use of antidiabetic medication.

### Statistical analysis

Continuous values were reported as means ± standard deviation (SD) or median [Q1, Q3], and compared using the Kruskal-Wallis test or the one-way analysis of variance. Categorical variables were reported as counts and percentages and compared by the χ2 test. The FGF19 levels showed a log-normal distribution (Supplemental Figure S2), so we applied a natural logarithmic transformation to the data before analysis. The correlation between ln-transformed FGF19 levels and metabolic parameters (FBG, HbA1c, BMI, WC, and hs-CRP) was tested using Pearson correlation. Linear regression was used to estimate the relationship between ln-transformed FGF19 levels and metabolic parameters. We utilized logistic regression to examine the relationship between ln-transformed FGF19 levels and diabetes, fitting three distinct models. Model 1 was adjusted for age and sex. Model 2 was adjusted for age, sex, BMI and WC. Model 3 was further adjusted for common diabetes risk factors, including smoking status, alcohol consumption, systolic and diastolic BP, total cholesterol, triglycerides and high-density-lipoprotein cholesterol. To assess collinearity, we tested all the models using the variance inflation factor (VIF). With VIFs less than 5 (data not shown), our results indicate the absence of collinearity among the variables. The shape of the relationship between ln-transformed FGF19 levels and diabetes was assessed using restricted cubic spline analysis, with three knots at quartiles 25th, 50th, and 75th. Subgroup analyses were performed by age (< 50, ≥ 50 years), sex (men, women), obesity (yes, no) and central obesity (yes, no). Likelihood-ratio test was used to test for modifications and interactions. Mediation analysis was conducted to explore the mediating effect of BMI on the relationship between ln-transformed FGF19 levels and diabetes. All analyses were conducted using R 4.2.1. A p-value < 0.05 was considered statistically significant for all analyses.

## Results

### Participant characteristic

Table 1 summarizes the baseline characteristics of the study population. This study comprises 1018 participants [555 (54.5%) female; mean age, 45.1 ± 10.1 years], 77 (7.56%) of whom had diabetes. Significant differences in age, BP, BMI, WC, and lipid profile were observed between diabetic and non-diabetic participants (all *P*-values < 0.05).

**Table 1:** Baseline characteristic of the study population

	Overall	Non-diabetes	Diabetes	*P*-value
	1018	941	77	
Age, year	45.10 ± 10.13	44.55 ± 9.89	51.81 ± 10.69	< 0.001
Female (%)	555 (54.5)	518 (55.0)	37 (48.1)	0.286
Current smoker (%)	97 (9.5)	90 (9.6)	7 (9.1)	1
Alcohol drinker (%)	50 (4.9)	44 (4.7)	6 (7.8)	0.346
Systolic BP, mmHg	119.23 ± 16.16	118.63 ± 16.00	126.60 ± 16.29	< 0.001
Diastolic BP, mmHg	78.49 ± 10.48	78.26 ± 10.55	81.25 ± 9.24	0.016
Body mass index, kg/m²	23.66 ± 3.56	23.50 ± 3.52	25.66 ± 3.52	< 0.001
< 24	584 (57.4)	559 (59.4)	25 (32.5)	
24–27.9	338 (33.2)	307 (32.6)	31 (40.3)	
≥ 28	96 (9.4)	75 (8.0)	21 (27.3)	
Waist circumference, cm	89.84 ± 15.41	89.54 ± 15.71	93.61 ± 10.50	0.026
HbA1c, %	5.80 ± 0.74	5.67 ± 0.38	7.35 ± 1.67	< 0.001
Fasting blood glucose, mmol/L	4.65 ± 1.14	4.47 ± 0.53	6.84 ± 2.95	< 0.001
Ln FGF19, pg/mL	5.60 ± 0.79	5.61 ± 0.80	5.46 ± 0.65	0.098
FGF19, pg/mL	250.21 [167.76, 396.05]	254.33 [168.84, 399.45]	218.19 [144.80, 346.33]	0.129
Hs-CRP, mg/L	0.76 [0.45, 1.41]	0.73 [0.43, 1.32]	1.34 [0.77, 2.29]	< 0.001
Total cholesterol, mmol/L	4.92 ± 0.89	4.90 ± 0.87	5.15 ± 1.06	0.021
Triglyceride, mmol/L	1.14 [0.81, 1.68]	1.12 [0.80, 1.62]	1.53 [1.08, 2.38]	< 0.001
HDL-Cholesterol, mmol/L	1.38 ± 0.30	1.39 ± 0.30	1.30 ± 0.26	0.013
LDL-Cholesterol, mmol/L	2.73 ± 0.73	2.72 ± 0.74	2.88 ± 0.71	0.059
Hypertension (%)	212 (20.8)	187 (19.9)	25 (32.5)	0.013
Antihypertensive medication (%)	63 (6.2)	52 (5.5)	11 (14.3)	0.005

Ln, natural logarithm. Continuous variables are expressed as mean ± standard deviation or as median [IQR]. Categorical variables are expressed as number (percent). HbA1c, hemoglobin A1c, hs-CRP, high sensitivity C-reactive protein

### Association between ln-transformed FGF19 levels and FBG, HbA1c, BMI and WC

Pearson correlation analyses between ln-transformed serum FGF19 and metabolic indicators are presented in Supplementary Figure S3. Ln-transformed FGF19 was not significantly correlated with FBG (*R* = 0.022, *p* = 0.49) or HbA1c (*R* = − 0.01, *p* = 0.74). Similarly, no significant correlations were observed with BMI (*R* = − 0.051, *p* = 0.10), waist circumference (*R* = 0.0032, *p* = 0.92), or ln-transformed hs-CRP (*R* = − 0.0042, *p* = 0.89). Linear regressions between ln-transformed FGF19 levels and FBG, HbA1c, BMI, WC, and ln-transformed hs-CRP are presented in Table 2. After adjusting for age and sex, BMI was inversely associated with ln-transformed FGF19 levels [β (95% Confidence Interval), -0.30 (-0.58, -0.03)], and there was no association between ln-transformed FGF19 levels and WC, FBG, HbA1c, and ln-transformed [β (95% Confidence Interval), -0.37 (-1.54, 0.80), 0.01 (-0.08, 0.09), -0.04 (-0.09, 0.02), and − 0.02 (-0.09, 0.05) respectively].

**Table 2:** Association of serum Ln FGF19 levels with body mass index, waist circumference, fasting blood glucose, HbA1c, and Ln hs-CRP

	β (95%CI)	
	Univariate	Multivariate*
Body mass index	-0.23 (-0.51, 0.05)	-0.30 (-0.58, -0.03)
Waist circumference	0.06 (-1.14, 1.27)	-0.37 (-1.54, 0.80)
Fasting blood glucose	0.04 (-0.05, 0.12)	0.01 (-0.08, 0.09)
HbA1c	-0.01 (-0.07, 0.05)	-0.04 (-0.09, 0.02)
Ln hs-CRP	0 (-0.07, 0.07)	-0.02 (-0.09, 0.05)

Ln, natural logarithm. HbA1c, hemoglobin A1c, hs-CRP, high sensitivity C-reactive protein.* Adjusted for age and sex

### Association between Ln-transformed Ln FGF19 and diabetes

**Fig. 1:**
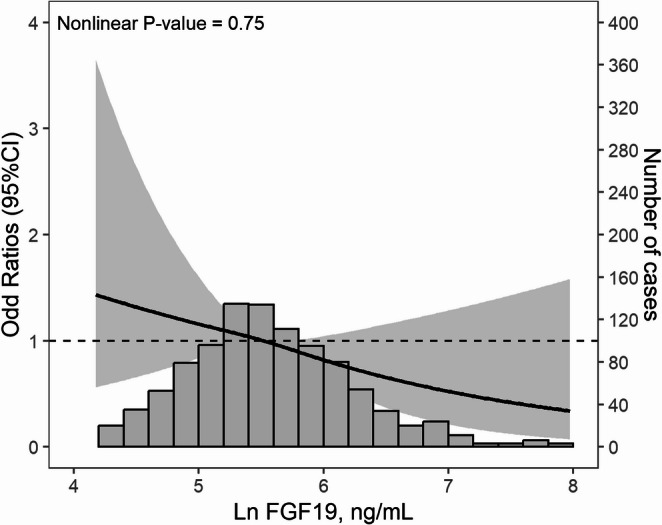
Restrict cubic spline of serum ln FGF19 levels and odds of diabetes. Ln, natural logarithm. The histograms below show the distribution of ln FGF19 concentration in the population. Model was adjusted for age, sex, current smoking, alcohol consumption, systolic blood pressure, diastolic blood pressure, body mass index, waist circumference, total cholesterol, triglyceride and high-density-lipid cholesterol

The logistic regression between ln-transformed FGF19 levels and diabetes is presented in Table 3. Elevated ln-transformed FGF19 levels were significantly associated with decreased risk of diabetes [Odds ratio (95% Confidence Interval), 0.66 (0.47, 0.95)] in a fully adjusted model (adjusted for age, sex, current smoking, alcohol consumption, systolic BP, diastolic BP, BMI, WC, total cholesterol, triglyceride and high-density-lipid cholesterol). Figure 1 displays the shape of the association between ln-transformed FGF19 levels and the risk of diabetes, with a monotonic relationship observed in the restricted cubic spline (P-value for nonlinearity = 0.75).

**Table 3:** Association between serum Ln FGF19 levels and diabetes

		Odd ratio (95% confidence interval)	*P*-value
Ln FGF19	Model 1	0.66 (0.46, 0.93)	0.001
	Model 2	0.70 (0.50, 1.00)	0.040
	Model 3	0.66 (0.47, 0.95)	0.025

Ln, natural logarithm.Model 1, adjusted for age and sex. Model 2, adjusted for age, sex, body mass index and waist circumference. Model 3, adjusted for age, sex, current smoking, alcohol consumption, systolic blood pressure, diastolic blood pressure, body mass index, waist circumference, total cholesterol, triglyceride, and high-density-lipid cholesterol

### Subgroup analysis

The subgroup analysis in Supplemental Table S1 shows that no interaction of ln-transformed FGF19 levels with age, sex, obesity and central obesity on diabetes was observed (all P-values for interaction > 0.05).

### Mediation analysis

**Fig. 2:**
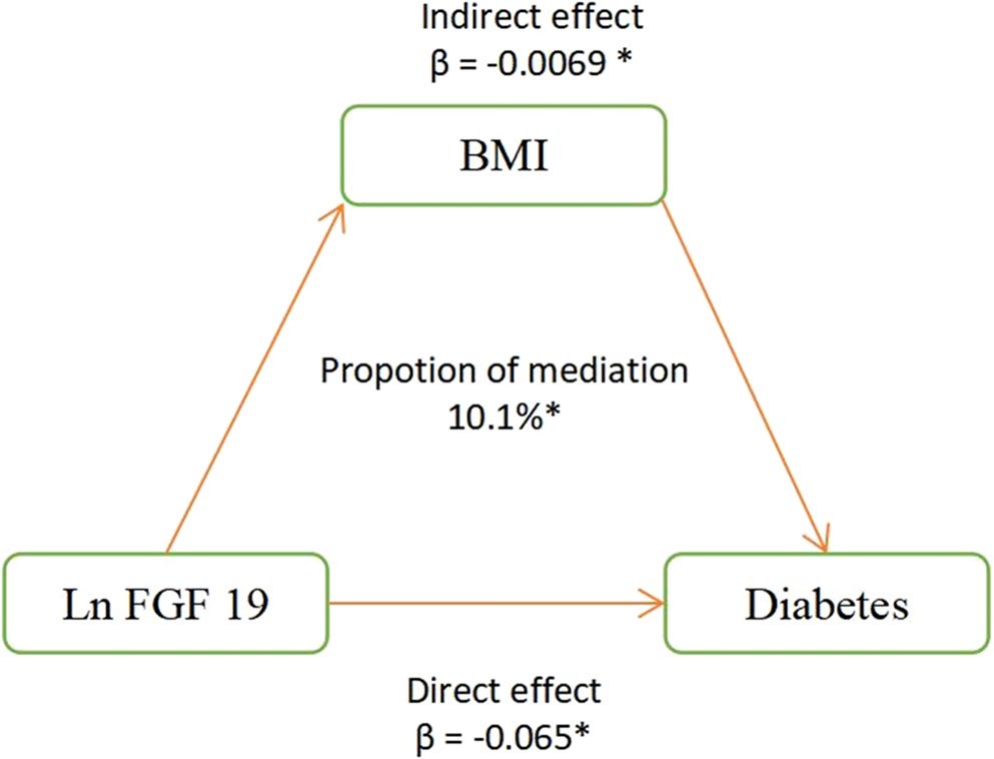
Mediation analysis of the association between ln FGF19 and risk of diabetes. Ln, natural logarithm. * *P*-value < 0.05

The mediation effect of BMI on the association between ln-transformed FGF19 levels and diabetes is presented in Fig. 2. The association between ln-transformed FGF19 levels and diabetes was partially mediated by BMI, with the proportion of 10.1% (P-value < 0.05).

## Discussion

Our study adds valuable insights to the existing body of literature on the relationship between FGF19, obesity, and diabetes in the Chinese population. Specifically, serum FGF19 levels negatively associated with odds of diabetes, and this relationship is about 10% mediated by the reduction of BMI.

One previous study indicated FGF19 levels were significantly lower in subjects with impaired fasting glucose and type 2 diabetes than in those with normal glucose tolerance [9]. We did not observe a significant inverse association between FGF19 and FBG, possibly due to the lower diabetes prevalence in our cohort (~ 8% vs. ~ 28%) and differences in exposure modeling (continuous FBG in our study vs. categorized glycemic groups in the prior study). However, our study confirmed the negative relationship between FGF19 and diabetes with a much larger sample size, which enhanced the statistical power and generalizability. Moreover, we conducted subgroup analysis and found the relationship remained consistent regardless of age, sex, and obesity and central obesity status, reinforcing the robustness of our findings. However, several subgroups included few diabetes cases (e.g., 21 cases among 96 participants with obesity), resulting in wide confidence intervals and limited power to detect interaction effects. Therefore, these subgroup findings should be interpreted cautiously, and larger studies—particularly among individuals with obesity—are warranted. Apart from diabetes, serum FGF19 level was also negatively associated with diabetes-related complications, such as diabetic retinopathy, diabetic nephropathies, subclinical atherosclerosis and polycystic ovary syndrome [10–13]. In addition, reduced fasting serum FGF19 levels have been observed in patients with inflammatory bowel disease, indicating a potential link between FGF19 and oxidative stress, inflammation, and immune response [14, 15], all of which contribute to the pathogenesis of diabetes. Our study not only demonstrated the association between FGF19 and diabetes but also utilized a mediation analysis that allowed us to fully investigate the relationships between FGF19, BMI, and diabetes. We found an inverse association between BMI and serum FGF19 levels, even after adjusting for age and sex, which was consistent with previous studies [4, 16]. Moreover, we discovered that BMI acted as a mediator in the relationship between diabetes and serum FGF19 levels, highlighting the multifactorial etiology of diabetes, wherein obesity and overweight have a crucial role in its progression by influencing the insulin sensitivity, metabolic pathways, or inflammation [17].

The mechanism linking FGF19, BMI, and diabetes is a complex interplay involving various biological processes. In animal models, FGF-19 signals through the FGF receptor 4 (FGFR4) and β-Klotho co-receptor, activation of this pathway suppresses bile acid synthesis and upregulates genes related to bile acid transport and detoxification. Bile acids also act as endocrine signaling molecules [e.g., through farnesoid X receptor (FXR) and Takeda G protein-coupled receptor 5 (TGR5)] that influence hepatic gluconeogenesis, glycogen synthesis, and incretin secretion [18]. Alterations in the bile acid pool and signaling tone represent a plausible medium-sized pathway between circulating FGF19 and diabetes risk [19]. Consistent with this, prior work suggests that impaired FGF19 signaling and disturbed bile acid profiles are observed in insulin-resistant states, and that modulating the FXR–FGF19 axis can affect glucose and lipid metabolism [20]. Therefore, part of the observed inverse association between FGF19 and diabetes may reflect downstream effects on bile acid composition and receptor-mediated metabolic signaling, which were not directly measured in our dataset. In parallel, FGF19 has been implicated in energy expenditure and body weight regulation [21]. Experimental studies indicate that central or peripheral FGF19 administration can reduce food intake and body weight and increase energy expenditure [5, 18], and FGF19 signaling has been linked to adipose tissue thermogenic programming (“browning”) that may improve insulin sensitivity [22]. These mechanisms align with our mediation finding that BMI explained a modest proportion (10.1%) of the FGF19–diabetes association, suggesting that adiposity-related pathways contribute, but do not dominate, the relationship. The relatively small mediation proportion also points to additional non-adiposity mechanisms. One candidate is metabolic inflammation. Chronic low-grade inflammation contributes to insulin resistance and β-cell dysfunction, and inflammatory cytokines such as Interleukin-6 (IL-6) and Tumor Necrosis Factor-α (TNF-α) may be more proximal mediators than hs-CRP, which is a downstream acute-phase reactant and may be less sensitive to subtle pathway changes in relatively young or community-based samples [23, 24]. In our cohort, hs-CRP was not associated with FGF19, so mediation by hs-CRP is not supported. Future work should consider broader inflammatory panels and tissue-specific markers to evaluate whether FGF19 relates to diabetes risk through inflammatory signaling independent of adiposity. Notably, clinical translation remains nuanced. In a phase 2 randomized controlled trial in patients with type 2 diabetes, aldafermin (NGM282), an engineered FGF19 analog, did not reduce plasma glucose or HbA1c, although modest weight reduction was observed at higher dose [7]. This trial indicated that the dysfunction of β-cell or islet is the predominant factor in hyperglycemia that cannot be corrected by weight loss, which is consistent with our study that the relationship between FGF19 and diabetes was to a small degree moderated by BMI reduction. Together, these data support our observation that BMI only partially mediates the FGF19–diabetes association and highlight the need for future longitudinal and mechanistic studies integrating bile acid metabolomics (total and individual bile acids), FXR/TGR5 pathway biomarkers, gut microbiome features, and comprehensive inflammatory profiling to delineate causal pathways and identify potential therapeutic targets.

### Strengths and limitations

The strength of this study lies in its large sample size and the use of mediation analysis to elucidate the underlying mechanisms of the relationship between FGF19 and diabetes. The study recruited participants from different communities in Shenzhen, a city known for its migrant population. As a result, the study sample may offer a representation of the population in China, at least in regard to ethnic origin and genetics. The partial mediation of this relationship by BMI reduction suggests that weight management may be an important strategy for improving FGF19 levels and reducing diabetes risk. It also suggests that interventions targeting multiple factors, such as FGF19 levels, body weight, and other lifestyle factors, may be more effective in addressing diabetes than interventions targeting a single factor alone.

Several limitations should be noted. First, because this study used a cross-sectional design, temporality cannot be established and causal inference is limited; reverse causation is also possible (e.g., dysglycemia, insulin resistance, or diabetes treatment may influence circulating FGF19 concentrations). To strengthen causal inference, future research should prioritize prospective cohort studies that track longitudinal changes in FGF19 and key metabolic traits to determine whether baseline FGF19 levels and/or within-person changes in FGF19 predict incident diabetes and trajectories of glycemic deterioration. In addition, Mendelian randomization analyses using genetic instruments for circulating FGF19 (or variants in the FXR–FGF19–FGFR4/β-Klotho pathway) could help clarify potential causal effects while reducing confounding and reverse causality. Second, although we adjusted for a broad range of covariates, residual confounding may persist because diabetes is a multifactorial and heterogeneous disease. Third, we did not distinguish between different types of diabetes (e.g., type 1 diabetes, type 2 diabetes, or other specific forms), and the association between FGF19 and diabetes may differ by diabetes subtype; future studies with detailed phenotyping and subtype classification are warranted.

## Conclusion

Our study discovers a monotonic and negative relationship between serum FGF19 levels and diabetes in the Chinese population, and this relationship is partially mediated by the reduction of BMI.

## Supplementary Information

Below is the link to the electronic supplementary material.

Supplementary File 1 (DOCX 587 KB)

## Data Availability

Data are available on request from the corresponding author.
