# Supplementary material for: Body mass index mediates the association between serum fibroblast growth factor-19 and diabetes
Source: J Diabetes Metab Disord. 2026 Jan 8;25(1):27. doi: 10.1007/s40200-025-01850-y (PMC12783406; doi:10.1007/s40200-025-01850-y)
Supplement: Supplementary file 1 — (DOCX 587 KB) [file 40200_2025_1850_MOESM1_ESM.docx]

**Table S1. Subgroup analysis of the relationship between serum ln FGF19 levels and risk of diabetes**

|  | Cases/total | Odd ratio (95%CI) | P-interaction |
| --- | --- | --- | --- |
| Age, years |  |  | 0.12 |
| <50 | 34/682 | 0.97 (0.59, 1.58) |  |
| ≥50 | 43/336 | 0.56 (0.34, 0.91) |  |
| Sex |  |  | 0.80 |
| Men | 40/463 | 0.70 (0.43, 1.14) |  |
| Women | 37/555 | 0.63 (0.38, 1.06) |  |
| Obesity |  |  | 0.82 |
| No | 56/922 | 0.65 (0.44, 0.98) |  |
| Yes | 21/96 | 0.72 (0.33, 1.58) |  |
| Abdominal obesity |  |  | 0.35 |
| No | 30/551 | 0.79 (0.48, 1.30) |  |
| Yes | 47/467 | 0.57 (0.34, 0.93) |  |

Ln, natural logarithm. FGF19, fibroblast growth factor-19. CI, confidence interval.

Model was adjusted for age, sex, current smoking, alcohol consumption, systolic blood pressure, diastolic blood pressure, body mass index, waist circumference, total cholesterol, triglyceride, and high-density-lipid cholesterol.

**Figure S1.** Flow chart of the study population

**
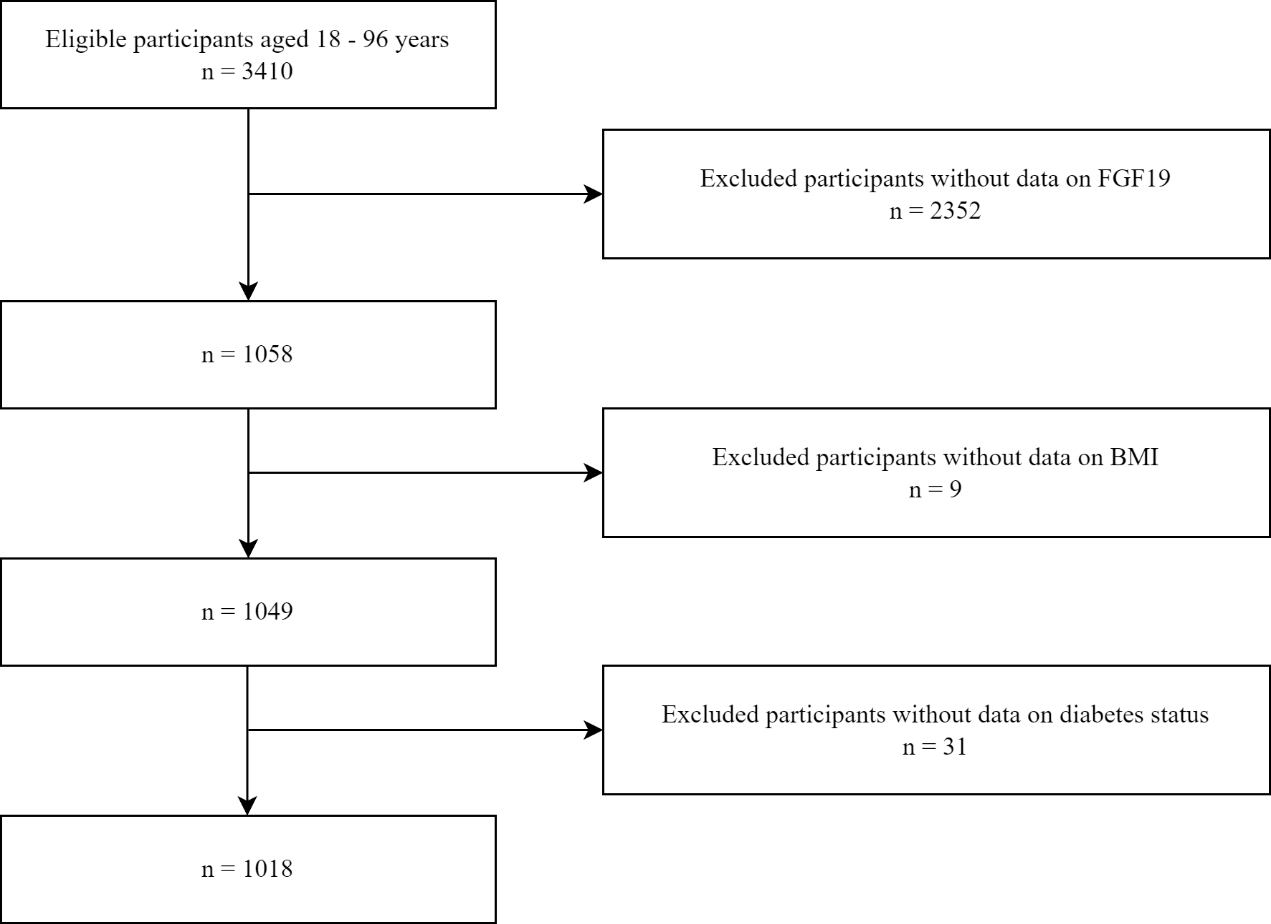
**

FGF19, fibroblast growth factor-19. BMI, Body mass index.

**Figure S2. Distribution and normal Q-Q plots of serum FGF19 levels and serum ln FGF19 levels**

**
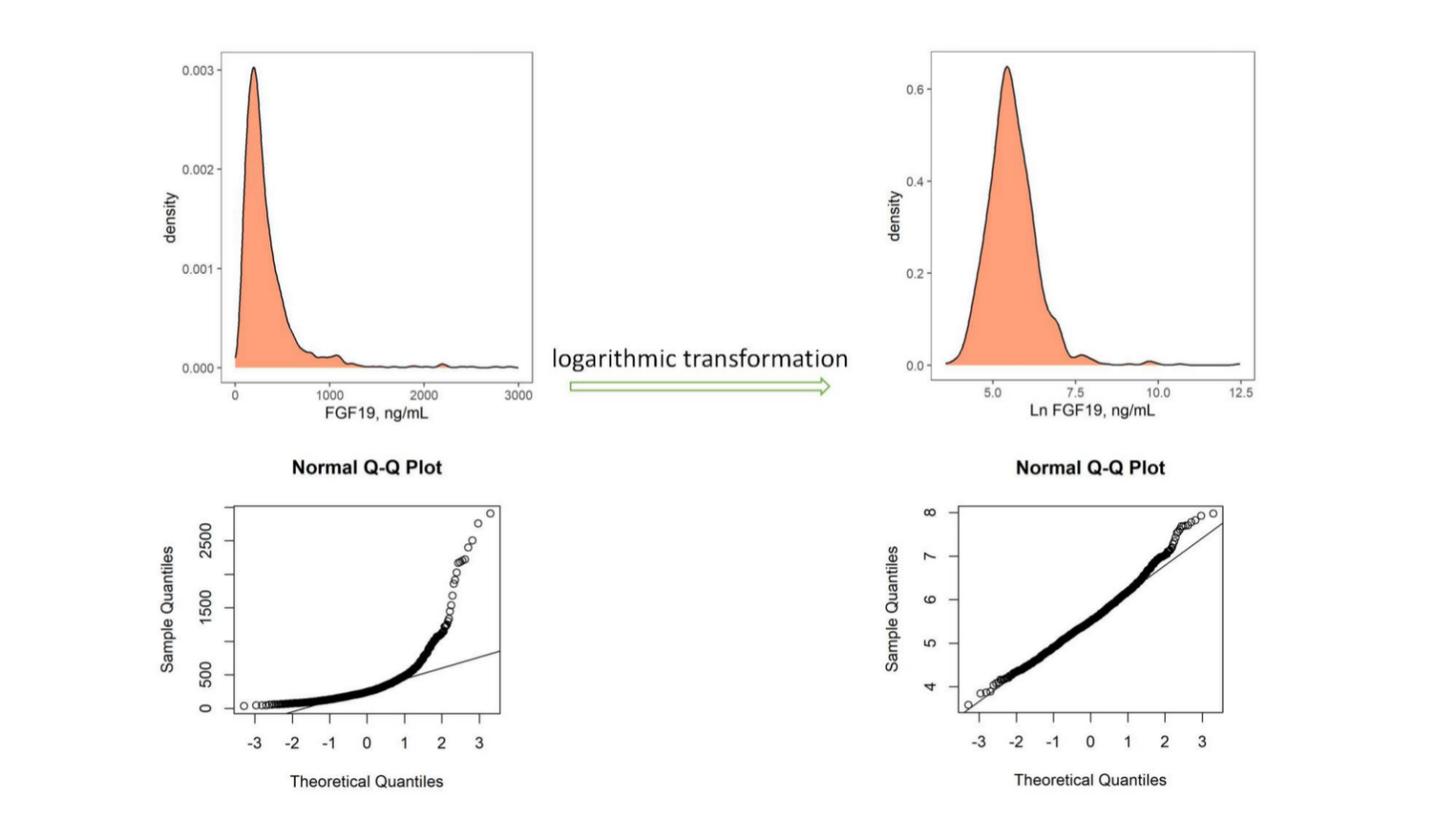
**

Ln, natural logarithm**.** FGF19, fibroblast growth factor-19.

**Figure S3. Pearson correlation between ln FGF19 concentration and fasting blood glucose, HbA1c, body mass index, and waist circumference**


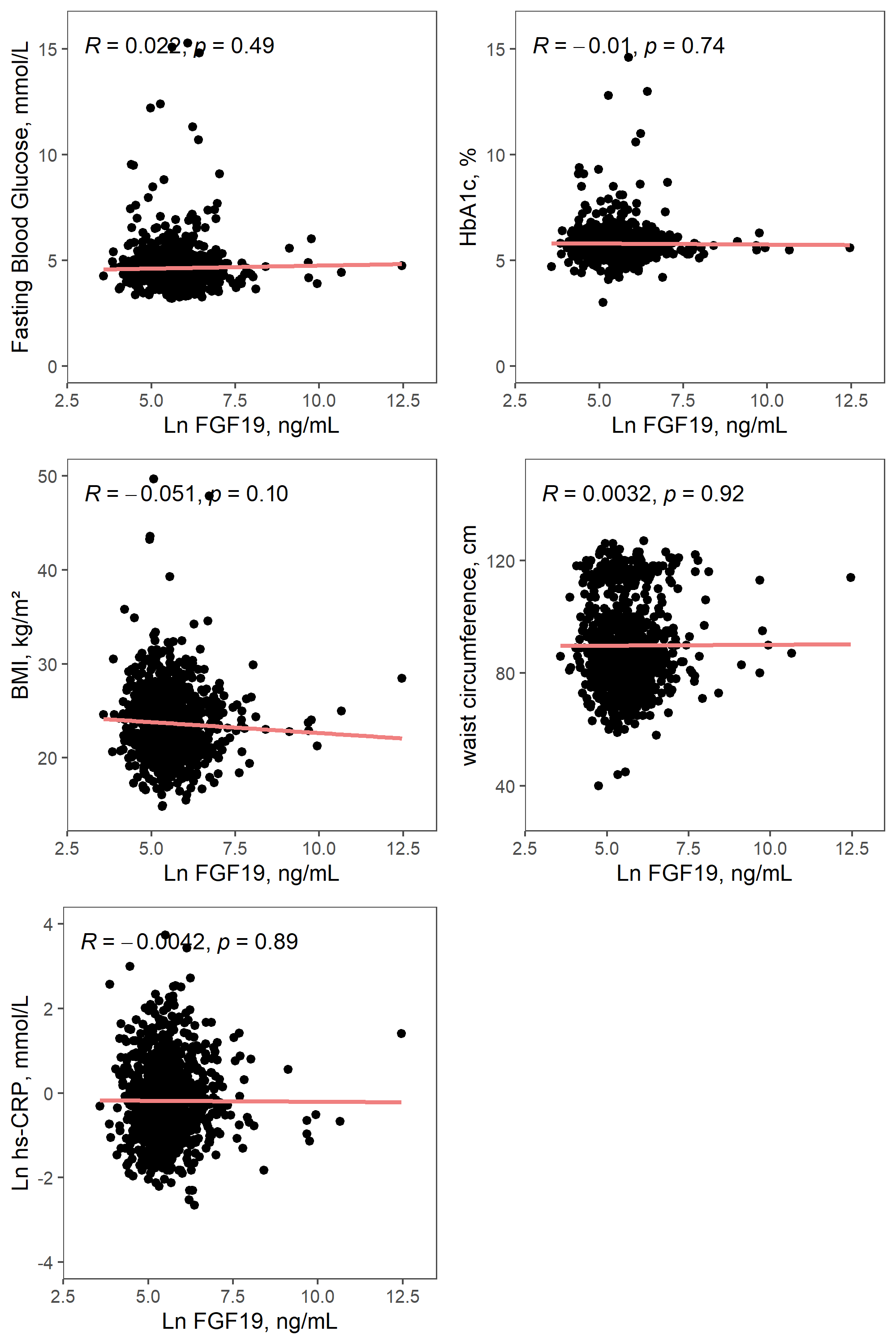
 Ln, natural logarithm, BMI, Body mass index. FGF19, fibroblast growth factor-19.
